# Cardiac Remodelling After Transcatheter and Surgical Aortic Valve Replacement: A Tough Call

**DOI:** 10.1093/icvts/ivag203

**Published:** 2026-07-30

**Authors:** Bianca Profire, Victoria Delgado

**Affiliations:** iCor Hospital University Germans Trias i Pujol, Carretera del canyet, Badalona, 08916, Spain; Grigore T. Popa University of Medicine and Pharmacy, Str.Universității 16, Iași, 700115, Romania; iCor Hospital University Germans Trias i Pujol, Carretera del canyet, Badalona, 08916, Spain; Institute of Research Germans, Trias i Pujol, Ctra de Can Ruti, Camí de les Escoles , Badalona, 08916, Spain

Across high-, intermediate-, and low-risk populations, there is extensive evidence indicating that transcatheter (TAVR) and surgical (SAVR) aortic valve replacement for severe aortic stenosis (AS) achieve broadly similar rates of survival and disabling stroke. However, the two strategies differ in their specific adverse events: paravalvular leak, vascular complications, and permanent pacemaker implantation are more frequent after TAVR, whereas severe bleeding, acute kidney injury, and new-onset atrial fibrillation (AF) occur more often after SAVR.^1^ In terms of the effects of TAVR and SAVR on cardiac remodelling, the available literature is less extensive. The relief of valvular obstruction by either SAVR or TAVR is generally followed by measurable reverse cardiac remodelling. Large comparative echocardiographic data show early and marked reductions in transvalvular gradients, modest improvement in left ventricular ejection fraction (LVEF), and recovery of LV longitudinal strain (LV-GLS) after both procedures, whereas right ventricular (RV) longitudinal function appears to follow a more divergent trajectory, with better preservation after TAVR but persistent impairment after SAVR.^2^ In addition, left atrial volume index (LAVI) and function improved after TAVR.^3^

The association of these cardiac remodelling changes with clinical outcomes has been evaluated in landmark randomized studies. In the Placement of Aortic Transcatheter Valves (PARTNER 3) trial, TAVR and SAVR showed similar valve haemodynamics, LV mass regression, and LVEF improvement at 1 year, while SAVR was associated with deterioration in RV systolic function and tricuspid regurgitation.^4^ Importantly, impaired tricuspid annular plane excursion (TAPSE) was associated with increased incidence of the composite outcome of death, stroke, or rehospitalization at 1 year of follow-up.^4^ In the Evaluation of TAVR Compared to Surveillance for Patients With Asymptomatic Severe Aortic Stenosis (EARLY-TAVR) trial, patients undergoing TAVR presented improvements in LV-GLS, regression in LV mass index and reduction in LAVI at 2 years of follow-up.^5^ Particularly, patients with normalization of those echocardiographic parameters presented lower rates of death, stroke, or unplanned cardiovascular hospitalization as compared to patients who did not exhibit such favourable remodelling. However, in this cohort of patients with asymptomatic severe AS, there was no SAVR arm to compare with.^5^

In the current issue of the Journal, Kislitsina et al^6^ provide new evidence on the impact of SAVR and TAVR on cardiac remodelling and its association with postoperative outcomes. A total of 373 patients with severe AS undergoing either TAVR (*n* = 195) or SAVR (*n* = 178) were evaluated with echocardiography at baseline and 1-year follow-up. Patients undergoing TAVR were older and had a greater burden of comorbidities, including AF, coronary artery disease, diabetes, hypertension, and cerebrovascular disease. Baseline LVEF, LV-GLS, RV-GLS, and RV free-wall strain were similar between groups, whereas patients treated with TAVR had larger LAVI and lower TAPSE than their counterparts. At 1 year of follow-up, LVEF and LV-GLS were not significantly different between treatment groups, although LV-GLS tended to improve more after SAVR as compared to TAVI. LAVI decreased modestly after both procedures, with similar absolute changes (−2.2 vs −3.2 mL/m^2^), although follow-up LAVI remained larger after TAVR. By contrast, RV function was preserved after TAVR but deteriorated after SAVR, as reflected by a significantly greater reduction in TAPSE (−0.2 vs −4.3 mm, *P *< .001), with a similar trend for RV free-wall strain (−0.1 vs +1.9%, *P *= .052). Although patients in the PARTNER 3 trial had higher baseline and follow-up LVEF,^4^ the present study provides consistent results: LVEF remained preserved and similar between TAVR and SAVR at 1 year, whereas TAPSE was preserved after TAVR but declined after SAVR. Nevertheless, it should be acknowledged that the use of TAPSE as the only marker of RV function after SAVR is limited by the angulation of the M-mode, and it does not represent the entire function of a cardiac chamber with a specific crescent shape geometry. The concept of postoperative “pseudo-dysfunction” remains an area of ongoing investigation and interpretation rather than an established consensus that invalidates the use of TAPSE in all postoperative settings.

Kislitsina et al^6^ also provide information on the association between cardiac remodelling and clinical outcomes. Their prognostic findings can be grouped into three chamber-specific directions. First, LAVI was one of the most consistent markers, with higher preoperative and postoperative values associated with mortality and AF recurrence, whereas greater reductions in LAVI after valve replacement were associated with lower stroke risk. Regarding LV remodelling, worse postoperative LV-GLS was associated with mortality and AF recurrence. RV parameters were mainly associated with stroke, with preoperative RV-GLS and TAPSE, as well as postoperative RV free-wall strain, RV-GLS, and TAPSE, associated with cerebrovascular events. These results contrast with previous studies such as the PARTNER 3 trial, where the prognostic signal related to cardiac chambers was mainly driven by RV systolic function, with impaired TAPSE associated with the composite outcome of death, stroke, or rehospitalization at 1 year.^4^ In the EARLY TAVR trial, abnormal integrated LV health, incorporating LV-GLS, LV mass index, and LAVI, was associated with higher event rates, and early TAVR resulted in more frequent preservation or normalization of LV health compared with clinical surveillance.^5^ The study by Kislitsina et al. provides incremental evidence by comparing both valve-replacement strategies and by linking LA, LV, and RV remodelling parameters to distinct clinical outcomes.

Currently, the recommendation of SAVR vs TAVR relies on the Heart Team discussion and shared decision-making in patients who are considered eligible for these therapies if they present severe AS, symptoms and/or reduction in LVEF.^1^ However, other echocardiographic parameters such as LAVI, LV mass, LV-GLS, or RV function are not included in the current recommendations. From the PARTNER trials, Généreux et al^7^^,^^8^ reported a larger proportion of patients improving in the extent of extravalvular cardiac damage (cardiac staging) after TAVR vs SAVR (59% vs 36%). These improvements were associated with greater improvements in quality of life and survival. The cardiac staging includes left atrial remodelling, RV dysfunction and pulmonary hypertension. Therefore, patients with larger extent of extravalvular cardiac damage could be preferably referred to TAVR to preserve or even improve cardiac remodelling since these improvements are also associated with better outcomes. Therefore, the article by Kislitsina et al. is incremental to the current evidence, highlighting the importance of characterizing cardiac remodelling after aortic valve replacement.
